# A Mobile Game for Patients With Breast Cancer for Chemotherapy Self-Management and Quality-of-Life Improvement: Randomized Controlled Trial

**DOI:** 10.2196/jmir.9559

**Published:** 2018-10-29

**Authors:** Hee Jun Kim, Sun Mi Kim, Heechul Shin, Joung-Soon Jang, Young In Kim, Doug Hyun Han

**Affiliations:** 1 Department of Internal Medicine College of Medicine Chung-Ang University Seoul Republic Of Korea; 2 Department of Psychiatry College of Medicine Chung-Ang University Seoul Republic Of Korea; 3 Department of Surgery College of Medicine Chung-Ang University Seoul Republic Of Korea

**Keywords:** mobile phone, breast cancer, chemotherapy, side effects, quality of life

## Abstract

**Background:**

The application of game-based learning in clinical practice has shown potential advantages in previous studies. However, there have been little efforts to use smartphone-based mobile games in the management of adult patients with cancer.

**Objective:**

The objective of our study was to evaluate if patient education using a mobile game may increase drug compliance, decrease physical side effects of chemotherapy, and improve psychological status in breast cancer patients.

**Methods:**

A total of 76 patients with metastatic breast cancer who were planned to receive cytotoxic chemotherapy were enrolled in this trial. Study participants were randomly assigned to a mobile game play group (game group, n=36) or a conventional education group (control group, n=40) in a ratio of 1:1. The patients were unblinded and followed prospectively for 3 weeks. Outcome measures included time spent for education, compliance to medication, physical side effects, and psychological side effects including quality of life (QoL).

**Results:**

Overall, 72 out of 76 patients completed the study after 3 weeks (95%). The subjects in the game group showed high levels of satisfaction with the app. The time spent playing the mobile game in the game group was longer than that spent for self-education in the control group (mean 22.2, SD 6.1 vs mean 5.5, SD 4.0 minutes a day; *P*<.001). The mobile game group showed better drug adherence (Korean version of the Medication Adherence Rating Scale; mean 7.6, SD 0.7 vs mean 6.5, SD 0.5; *P*<.001). The use of the mobile game was associated with lower rates of chemotherapy-related side effects, such as nausea, fatigue, numbness of hand or foot, and hair loss, than the control group. The game group exhibited better QoL during chemotherapy (mean 74.9, SD 3.5 vs mean 72.2, SD 5.3; *P*=.01). However, there were no significant differences in terms of depression and anxiety scales.

**Conclusions:**

This study suggests the feasibility and potentiality of the use of smartphone mobile games for patients with breast cancer receiving chemotherapy. Education using a mobile game led to better patient education, improved drug compliance, decreased side effects, and better QoL compared with conventional education. Mobile games can be used as easy, fun, and effective measures for patient education and have the potential to improve treatment outcomes.

**Trial Registration:**

ClinicalTrials.gov NCT03205969; http://clinicaltrials.gov/ct2/show/NCT03205969 (Archived by WebCite at http://www.webcitation.org/71jfSBOq9).

## Introduction

Playing console and Web-based games is a popular free-time activity among children, adolescents, and adults [1]. Recent studies with health-related internet games have shown positive effects, such as improving coping strategies for health problems, strengthening treatment compliance, and increasing motivation to overcome the difficult times during illness [2]. Potential benefits of using computer-based patient education programs include increased patient knowledge in perceived information competence [3]. Game-based learning may be enjoyable, interesting, and immersive and therefore, more effective than classical learning [4]. Patient education has shown to be effective in the management of patients with teenage cancer, diabetes, and asthma [2]. However, there have been limited studies conducted in adults.

Chemotherapy is the main treatment for breast cancer, which has proven to reduce the rate of recurrence and mortality in breast cancer patients [5]. Chemotherapy is accompanied by significant side effects. Consequently, many cancer survivors experience physical and psychological symptoms that hamper their quality of life (QoL) and disrupt their daily living activities, family relationships, and work schedules [6]. Diarrhea, nausea, vomiting, hair loss, and mucositis are among the most common side effects [7]. These side effects may cause poor drug compliance, prohibiting successful anticancer treatment. Poor education is one of the main determinants of poor adherence to chemotherapy [8]. Therefore, proper education and sufficient communication are important to increase adherence, which may eventually contribute to improved clinical outcomes [9,10].

An easily accessible, immersive, and interactive Web-based game (ILOVEBREAST) was developed for adult patients with metastatic breast cancer. Although previous efforts using games were mostly focused on children, adolescents, or young adults, ILOVEBREAST is specifically designed for adult users. This study was a proof-of-concept randomized controlled trial aimed at evaluating the benefits of smartphone-based mobile game use in breast cancer patients receiving cytotoxic chemotherapy. We hypothesized that mobile gaming would lead to increased drug compliance, decreased physical side effects of chemotherapy, and improved psychological status among patients.

## Methods

### Patients

Patients with pathologically proven, clinical stage IV breast cancer were enrolled in this study at Chung-Ang University Hospital, Korea, from September 2013 to September 2014. Patients who had metastatic breast cancer and agreed to participate in an education-controlled trial of mobile game management were screened with the research and the patient edition version of Structured Clinical Interview for Diagnostic and Statistical Manual of Mental Disorders, Fourth Edition, Text Revision Axis I Disorders [11]. The inclusion criteria were as follows: females diagnosed with metastatic breast cancer, aged 18-65 years, use of at least third-line palliative chemotherapy treatment comprising taxanes, anthracyclines, capecitabine, and platinum compounds, and ability to use a smartphone for the mobile game. The exclusion criteria were as follows: current or history of uncontrolled medical diseases except for breast cancer, psychiatric diseases, including major depressive disorder and anxiety disorders, and history of substance abuse, including alcohol, nicotine, and drugs. Major depressive disorder and anxiety disorder were diagnosed based on the Diagnostic and Statistical Manual of Mental Disorders, Fourth Edition, Text Revision, the authoritative guide to the diagnosis of mental disorders published by American Psychiatric Association [12].

The Chung-Ang University Hospital Institutional Review Board approved the research protocol for this study (Number C20141447). Informed consent was obtained from all patients during hospitalization for chemotherapy after explaining the design, protocol, and consequences of the study (NCT03205969).

### Description of the Mobile Game

**Figure 1:**
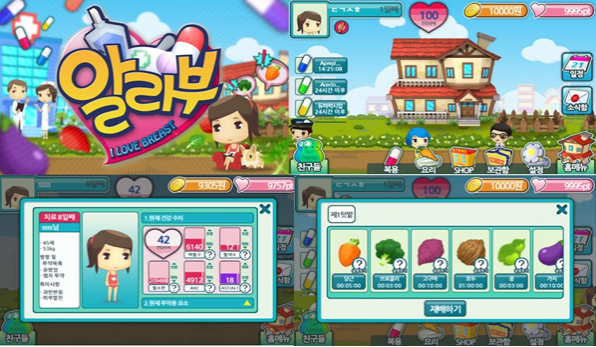
Representative screenshots of the ILOVEBREAST game. Source and copyright: Industry Academic Cooperation Foundation, Chung-Ang University Hospital.

A mobile game, ILOVEBREAST (CLGAMES, Seoul, Korea), was developed with an intention to improve self-management and to reduce the side effects of chemotherapy drugs. The ILOVEBREAST program used in this study was a 3-week program using typical multiplayer, social network, and platform-based features. The game’s key pedagogical features were as follows: education for preventing side effects of anticancer drugs, support for the prevention of side effects of anticancer drugs including numbness, hair loss, and loss of appetite, encouragement of mood and activity, including exercise, pet walking, cooking, and social game playing, which may facilitate participation in such activities in real life, and self-assessment using a personal avatar, as seen in Figure 1.

At the start of game play, an avatar is generated based on the patient’s medical information including the status of blood components, general medical condition, and chemotherapeutic drugs. Avatars are to visit their home, pharmacy, hospital, and gymnasium; make purchases from shops; and operate a farm to harvest ingredients for food. They can receive prescribed medications, cook food appropriate for their health, and exercise. Depending on the patient’s medication dosage, avatars can pursue a quest to minimize the side effects. The quest consists of taking the medication at the right time, cooking a meal for oneself, exercising, going outside for a walk, and chatting with a friend. An alarm alerts the avatar to take medications timely. Each time the avatar accomplishes a quest, “heart coins” are rewarded. The greater the number of the coins the patient receives, the greater the improvement in the avatar’s health status. The avatar can then use these coins to purchase items such as hats, gloves, and food ingredients. Patients are asked to visit their doctor weekly to assess their health status.

### Study Procedure

This study was a 3-week prospective trial. All study participants received a combination of 4 chemotherapy drugs (taxanes, anthracyclines, capecitabine, and cisplatin). Patients were randomly assigned to education using mobile game play (game group) or conventional education (control group) utilizing an interactive Web randomization system.

For patients in the game group, the study mobile game (ILOVEBREAST) was installed on the participants’ smartphones. They were recommended to play the game for >30 minutes a day, 3 times per week. They were interviewed every week via cell phone until the end of the study. The patients in the game group were checked with the record of access to the ILOVEBREAST game. The patients in the control group received routine care. A brochure elaborating the coping strategies for the side effects of chemotherapy drugs was provided to the control group only. The 26-page education material consisted of 2 parts. Part 1 provided the overall guidelines relating to life patterns, definition and purpose of chemotherapy, and broad side effects of chemotherapy. Part 2 contained individualized educational material comprising each patient’s purpose for chemotherapy, the name of the anticancer drugs, chemotherapy schedule, individualized management of side effects and symptoms, and guidance for daily life and mental attitude. The subjects in the control group were recommended to read the material for >30 minutes a day, 3 times per week. The patients in the game group were requested to rate the level of satisfaction using the following 8 questions:

1. What percentage of game content do you use while playing the game?
2. Is the game difficult to play?
3. Is the game fun?
4. Is the game helpful for taking your medication?
5. Does the game provide you with information about breast cancer and treatment?
6. Does the game decrease your unease with chemotherapy?
7. Do you plan to play ILOVEBREAST during your next chemotherapy session?
8. Would you recommend ILOVEBREAST to other patients with breast cancer?

The questions were assessed using a self-reported scale of 10 levels with 10 indicating “very bad” to 100 indicating “very good.”

Trial outcomes were measured in the following 4 domains: time spent for education, compliance to medication, physical side effects, and psychological side effects including QoL. Education time was measured as either the time spent for game playing or self-education using the brochure with preventive measures. The manufacturer (CLGAMES, Seoul, Korea) provided the time the users spent playing the mobile game. Medication compliance was assessed with the Korean version of the Medication Adherence Rating Scale (K-MARS), which has a Cronbach reliability alpha of .71 [13].

Physical and psychological side effects were assessed at baseline and the end of the 3-week follow-up period using questionnaires [7]. The questionnaires for the presence and severity of side effects (ie, nausea, fatigue, decreased appetite, numbness on hand or foot, stomatitis, diarrhea or constipation, hair loss, and skin rash) were measured using a 5-point Likert scale. Questionnaires for psychological assessment included the Beck Depression Inventory (BDI) with a Cronbach reliability alpha of .83 [14], the Spielberger State-Trait Anxiety Scale with alpha of .84 [15], and the World Health Organization Quality of Life-BREF Scale with alpha of .87 [16]. BDI is one of the most widely used self-reported scales to assess the severity of depressive mood and consists of 21 multiple-choice [14] questions. The Spielberger State-Trait Anxiety Scale has 20 items each to measure trait anxiety and state anxiety [17]. State anxiety means a temporary response to perceived threats, whereas trait anxiety refers to a consistent personality trait to experience anxiety. For this study, only state anxiety was measured because we intended to measure short-term response related to chemotherapy [15]. The World Health Organization Quality of Life-BREF Scale is an abbreviated generic Quality of Life Scale developed through the World Health Organization and consists of 26 items in the following 4 domains: physical health, psychosocial health, social relationships, and environment [16].

### Statistics

Because this study was a proof-of-concept trial, the sample size calculation was done on a practical basis. In the center where this study was undertaken, an average of 200 patients receives cytotoxic chemotherapy in a month. We assumed approximately 30 patients a month would meet the inclusion and exclusion criteria of this study and planned to recruit 90 patients for 3 months. Patient enrollment was slower than expected, and we decided to stop enrollment 1 year after the initiation of the study.

Continuous variables were presented as mean (SD), and categorical variables were presented as counts and percentages. Continuous variables were compared with independent *t* tests or Mann-Whitney U-tests as appropriate. The Chi-square test or Fisher’s exact test was used for dichotomous variables. For all statistical analyses, the significance level alpha was set at.05, and all analyses were performed using SPSS 18.0 (Chicago, IL, USA).

## Results

### Demographic Characteristics

**Figure 2:**
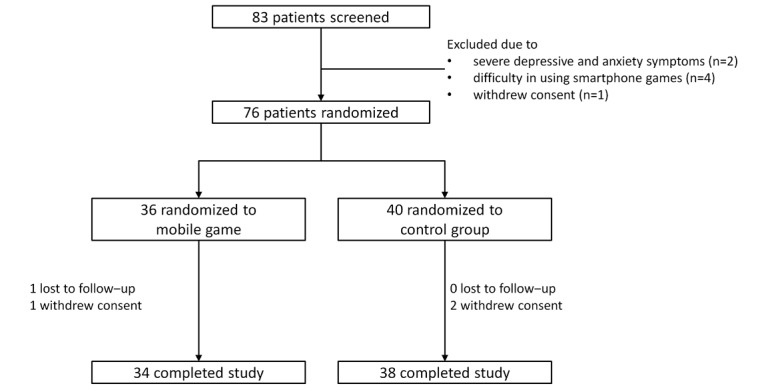
CONSORT study flow diagram.

A flow diagram of the study is shown in Figure 2. A total of 83 patients with metastatic breast cancer agreed to participate in the study. Among them, 2 were excluded because of severe depressive and anxiety symptoms, 4 for having difficulties in using the mobile game, and 1 for withdrawal of consent for an unspecified reason. Among the 76 female patients who were finally enrolled, 36 and 40 were randomly assigned to the mobile game and control groups, respectively. After 3 weeks of the study duration, 34 and 38 patients in each group completed the study (94.7%).

The baseline characteristics of the study subjects are summarized in Table 1. The mean age was 50.9 (SD 7.0) years, and all the patients were female. All the participants had an Eastern Cooperative Oncology Group performance status between 0 and 2. There were no significant differences in demographics status and socioeconomic status, pathologic classification, and hormonal status between the 2 groups.

### Overall Satisfaction

**Figure 3:**
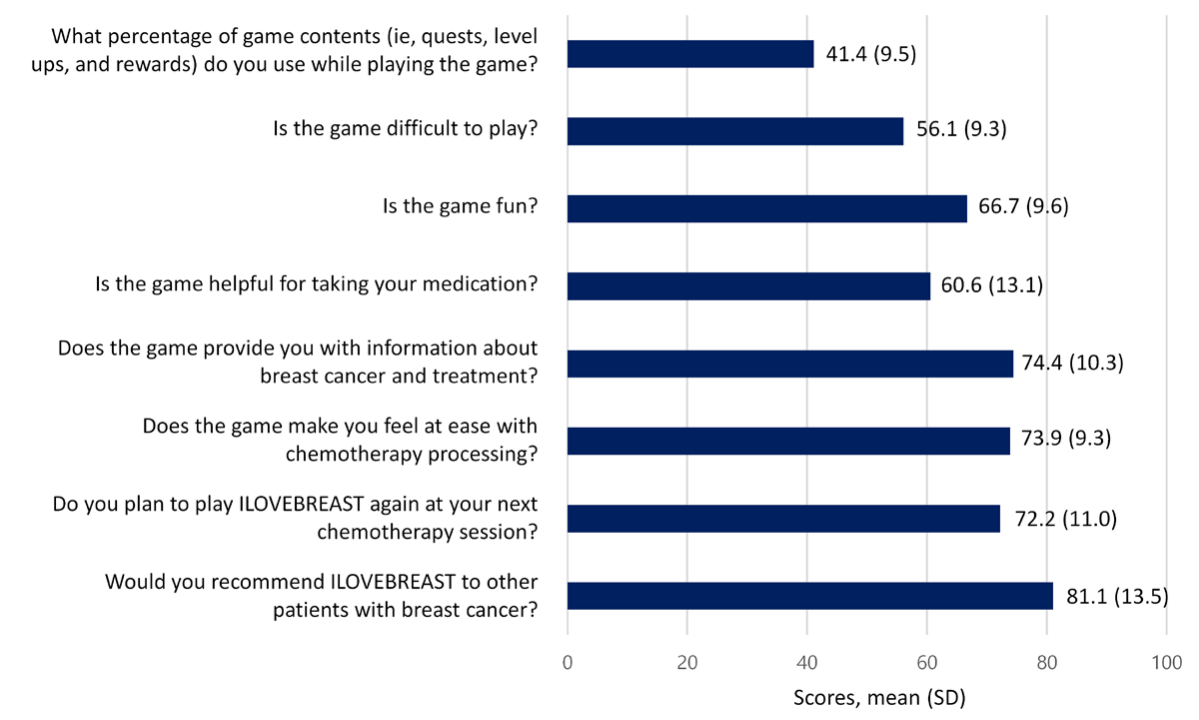
Levels of satisfaction in ILOVEBREAST.

The patients in the game group were requested to assess their level of satisfaction with ILOVEBREAST, as seen in Figure 3. Patients in the game group played 41% of the game contents (quests, level ups, and rewards), and more than half responded that it was difficult to use (20/36, 56%). However, 67% (24/36) and 61% (22/36) of the patients responded that the game was fun and helpful in taking their medications, respectively. Approximately ¾ of patients (27/36) responded positively in terms of acquisition of information (74.4%), usefulness in overcoming chemotherapy side effects (73.9%), and willingness to play again (72.2%). Furthermore, approximately 81% reported they would recommend the game to other patients with breast cancer.

**Table 1:** Demographic data.

Characteristics		Game group (n=36)	Control group (n=40)	*P* value
Age (years), median		49.8	52.1	.24
Years of education, mean (SD)		13.5 (2.0)	13.2 (1.9)	.51
**Economic status^a^, n (%)**				.94
	Highest tertile	2 (6)	2 (5)	
	Middle tertile	32 (89)	35 (88)	
	Lowest tertile	2 (6)	3 (8)	
Current smoker, n (%)		5 (14)	4 (10)	.43
Social drinking, n (%)		17 (47)	19 (48)	.49
History of mobile gaming, n (%)		20 (56)	22 (55.0)	.50
**Performance status, n (%)**				.94
	ECOG^b^ 0-1	35 (97)	39 (98)	
	ECOG 2	1 (3)	1 (2.5)	
	ECOG 3-4	0 (0)	0 (0.0)	
**Pathologic characteristics, n (%)**				.99
	Invasive ductal carcinoma	35 (97)	38 (95)	
	Others	1 (3)	2 (5)	
**Stage, n (%)**				.79
	Initial stage IV	8 (22)	10 (25)	
	Relapsed stage IV	26 (72)	30 (75)	
**Hormone receptor status, n (%)**				.77
	Positive	25 (69)	29 (73)	
	Negative	11 (31)	11 (28)	
**Human epidermal growth factor receptor 2, n (%)**				.07
	Positive	13 (36)	7 (18)	
	Negative	23 (64)	33 (83)	
**Triple negative phenotype, n (%)**				.84
	Nontriple negative	30 (83)	34 (85)	
	Triple negative	6 (17)	6 (15)	

^a^Economic status was classified into the following tertiles: highest, > US $100,000; middle, $30000-$100,000; and lowest, <$30000.

^b^ECOG: Eastern Cooperative Oncology Group.

### Study Endpoints

The time spent on game playing in the mobile game group was higher than that spent for self-education in the control group (22.2, SD 6.1 vs 5.5, SD 4.0 minutes; *P*<.001; Table 2). The game group also showed improved compliance to medications compared with the control group (K-MARS score, 7.6, SD 0.7 vs 6.5, SD 0.5; *P*<.001). The patients in the study group reported lower rates of physically adverse events, such as nausea (*P*=.02), fatigue (*P*=.02), and numbness in the hand or foot (*P*=.02). Clinically significant adverse events, defined by grade ≥3 of Common Terminology Criteria for Adverse Events 3.0, including nausea (*P*=.02), fatigue (*P*=.002), and hair loss (*P*=.01) was shown to be lower in the game group.

**Figure 4:**
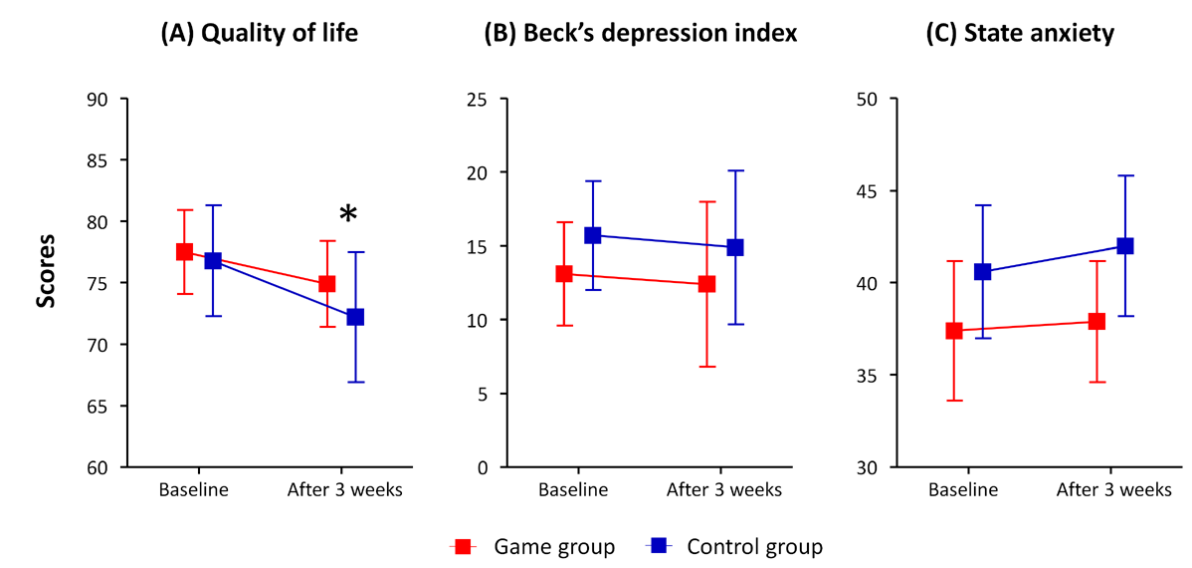
Psychological adverse events: (A) quality of life, (B) Beck’s depression index, and (C) state anxiety. *: P<.05.

After the study period of 3 weeks, the game group showed a higher QoL than in the control group, as seen in Table 3 and Figure 4. The decrease in the QoL score was also significantly lower in the game group than in the control group (*P*=.01). Regarding the subitems of QoL, the use of the mobile game was associated with lower decreases in physical health and environment but a higher decrease in psychological health than the usual care. There were no significant differences in the BDI score or state anxiety scale between the 2 groups.

**Table 2:** Comparison of physically adverse events between study groups.

Education time and medication compliance		Game group (n=34)	Control group (n=38)	*P* value
Time spent for game playing or self-education (minutes/day), mean (SD)		22.2 (6.1)	5.5 (4.0)	<.001
Korean Medication Adherence Rating Scale, mean (SD)		7.6 (0.7)	6.5 (0.5)	<.001
**Any physical adverse events, n (%)**				
	Nausea	29 (85)	23 (61)	.02
	Fatigue	16 (47)	29 (76)	.02
	Decreased appetite	16 (47)	11 (29)	.18
	Numbness of hand/foot	0 (0)	22 (58)	.02
	Stomatitis	0 (0)	4 (11)	.15
	Gastrointestinal (diarrhea or constipation)	7 (21)	9 (24)	.97
	Hair loss	0 (0)	10 (26)	.27
	Skin rash	0 (0)	0 (0)	N/A^a^
**Physical adverse events grade ≥3 of Common Terminology Criteria for Adverse Events, n (%)**				
	Nausea	5 (15)	0 (0)	.02
	Fatigue	1 (3)	12 (32)	.002
	Decreased appetite	3 (9)	6 (16)	.59
	Numbness of hand and foot	0 (0)	3 (8)	.28
	Stomatitis	0 (0)	3 (8)	.28
	Gastrointestinal (diarrhea or constipation)	1 (3)	5 (13)	.25
	Hair loss	0 (0)	8 (21)	.01
	Skin rash	0 (0)	0 (0)	N/A

^a^N/A: not applicable.

**Table 3:** Comparison of psychological side effects between study groups.

Psychological side effects	Baseline			At 3 weeks			Differences		
	Game, mean (SD)	Control, mean (SD)	*P* value	Game, mean (SD)	Control, mean (SD)	*P* value	Game, mean (SD)	Control, mean (SD)	*P* value
Quality of life	77.5 (3.4)	76.8 (4.5)	.48	74.9 (3.5)	72.2 (5.3)	.01	−2.6 (1.5)	−4.6 (4.4)	.01
Overall quality	2.3 (0.7)	2.3 (0.6)	.74	2.5 (0.7)	2.5 (0.6)	.85	−0.3 (0.3)	0.4 (0.2)	.65
Overall health	1.9 (0.5)	1.7 (0.6)	.14	2.1(0.7)	2.3 (0.6)	.47	0.3 (0.6)	0.6 (0.9)	.08
Physical health	19.1 (2.2)	18.7 (1.8)	.37	20.4 (2.2)	21.1 (1.7)	.16	1.3 (1.0)	2.4 (1.9)	.003
Psychological health	18.1 (1.4)	17.6 (43.1)	.31	18.7 (1.4)	19.3 (4.3)	.43	−2.1 (0.7)	1.7 (2.9)	.02
Social relationships	9.3 (1.3)	9.6 (1.6)	.37	9.4 (1.2)	9.9 (1.9)	.22	0.1 (0.3)	0.2 (2.1)	.67
Environment	23.8 (1.7)	22.9 (3.5)	.16	24.3 (1.9)	24.3 (3.3)	.90	0.4 (1.1)	1.4 (2.5)	.03
Beck’s depression index	13.1 (3.5)	12.4 (5.6)	.51	15.7 (3.7)	14.9 (5.2)	.50	2.6 (1.1)	2.6 (1.7)	.99
State anxiety	37.4 (3.8)	37.9 (3.3)	.43	40.6 (3.6)	42.0 (3.8)	.11	3.4 (0.9)	4.1 (3.4)	.21

## Discussion

### Principal Findings

The mobile game ILOVEBREAST was developed to help patients with advanced breast cancer learn more about the disease course, properties of medications, and expected adverse drug reactions. Adults users were generally satisfied with the game app. The mobile game-based intervention improved patient compliance, decreased the prevalence of physical side effects, and maintained the patients’ QoL compared with the conventional care. The use of the game had no impact on psychological side effects including mood and anxiety.

### Improved Drug Compliance

The patients in the study group used approximately 40% of the game contents, and the overall satisfaction was acceptable. Notably, a high proportion of the patients expressed a willingness to play the game again at their next chemotherapy session and to recommend the game to other patients. In addition, education time and drug adherence were significantly higher in the game group than in the control group. The quests for taking medication and reminder alarms in the game may have contributed to improving patients’ knowledge of the disease, medication, and adverse events. Previous studies have shown that knowledge about the disease course and adverse drug events is closely linked to patient drug adherence [18]. Since the release of the “Re-Mission” titles, which showed good adherence to treatments and easy access to knowledge, from HopeLab in California, the use of computer games in health education and physical education has been considered to have positive effects [19]. Some video games have also been used as vehicles to transmit health education regarding fire and street safety, and self-management of diabetes and asthma [20,21]. Although most health education games have focused on children, adolescents, and young adults [22-25], this study implies that adults can also utilize and benefit from game-based learning if the contents are specifically designed for them.

### Physical Side Effects and Quality of Life

The ILOVEBREAST game presents an avatar that can prevent side effects of numbness and hair loss by purchasing gloves or hats. The users are rewarded for continuous medication use, and they can chat with other players who have the same disease and difficulties. The player can buy food ingredients and learn how to cook and prepare healthy diets. We assume that such activities resulted in decreased prevalence and severity of the physical side effects in the game group. Twitter, as a complementary method, was shown to be effective in increasing knowledge about overall disease course, survivorship, cancer types and biology, and treatment options in patients with breast cancer in a previous study [26]. The Web-based game properties of immersivity, attention-maintaining properties of stories, engaging properties of interactivity, and behavior-change technology may improve health-related behaviors and habits in patients with breast cancer [27]. Several video games have been created to distract people from acute or chronic pain. The “Re-Mission” from HopeLab in California is the most representative mobile game that has these positive effects [19].

The mobile game group in this study showed a higher QoL in various domains, including total health, physical health, psychological health, and environmental areas. Pompeu et al reported improved QoL in 7 patients with Parkinson disease by a game named Kinect Adventures! (Xbox 360, Microsoft Game Studios, United States) [28]. Reichlin et al also reported an improvement in the QoL in 13 patients with localized prostate cancer after playing an interactive game, Time After Time (AMIGO, Gigamic, Piatnik, Playroom Entertainment, Germany) [29].

However, we observed no improvement in the psychological effects in this study. Chen et al reported that pediatric patients with obesity who played educational games for about 10 weeks-2 years improved their psychosocial functions, including depression, self-efficacy, and self-esteem [30]. One possible explanation is the short study duration (ie, 3 weeks) of this research. The age of the participants may be another factor. Unlike children and adolescents, older patients may have a lower propensity to have interest in playing a game.

### Study Limitations

The major limitation of this study is the small sample size and the short study period. Future studies are needed to confirm which patients most benefit from the strategy using mobile games and whether the benefits may lead to an improvement in hard endpoints such as mortality or recurrence. Next, adverse events related to chemotherapy were assessed subjectively. Objective assessments such as scales, blood tests, or laboratory tests may better describe the adverse effects of chemotherapy. Also, this study reflects the difficulty when using games for adult patients. Although the patients generally expressed satisfaction, the users experienced only a limited amount of the contents, and more than half had difficulty using the app. We believe this fact provides an important lesson for future developers.

### Conclusion

A mobile game, ILOVEBREAST, was helpful in educating adult patients with breast cancer receiving cytotoxic chemotherapy. The game was associated with improved drug compliance, decreased prevalence rates of physical side effects, and better QoL. Patient education with smartphone mobile games can be used as an easy, fun, and effective measure to promote treatment adherence, which may potentially lead to improved survival.

## Abbreviations

BDI: Beck Depression Inventory
K-MARS: Korean version of the Medication Adherence Rating Scale
QoL: quality of life

## Appendix

Multimedia Appendix 1 CONSORT-EHEALTH checklist ( V1.6.1).
