# Supplementary material for: A Mobile Game for Patients With Breast Cancer for Chemotherapy Self-Management and Quality-of-Life Improvement: Randomized Controlled Trial
Source: J Med Internet Res. 2018 Oct 29;20(10):e273. doi: 10.2196/jmir.9559 (PMC6305659; doi:10.2196/jmir.9559)
Supplement: Multimedia Appendix 1 [file jmir_v20i10e273_app1.pdf]

CONSORT-EHEALTH Checklist V1.6.2 Report

Manuscript Number 9559

(based on CONSORT-EHEALTH V1.6), available at [http://tinyurl.com/consort-ehealth-v1-6].

Date completed
10/21/2018 9:26:40
by
Han

A Mobile Game for Patients With Breast Cancer: Randomized Controlled Trial

TITLE

1a-i) Identify the mode of delivery in the title
We address "mobile" in our paper
1a-ii) Non-web-based components or important co-interventions in title
They were interviewed every week by cell phones
1a-iii) Primary condition or target group in the title
A Mobile Game for Patients With Breast Cancer: Randomized Controlled Trial

ABSTRACT

1b-i) Key features/functionalities/components of the intervention and comparator in the METHODS section of the ABSTRACT
Study participants were randomly assigned to a mobile game play group or a conventional education group in a ratio of 1:1. The patients were unblinded and followed prospectively for 3 weeks.
1b-ii) Level of human involvement in the METHODS section of the ABSTRACT
Our intervention was education through smart-phone based. So this comment was thought as slightly inappropriate.
1b-iii) Open vs. closed, web-based (self-assessment) vs. face-to-face assessments in the METHODS section of the ABSTRACT
The patients were unblinded and followed prospectively for 3 weeks
1b-iv) RESULTS section in abstract must contain use data
Overall, 72 out of 76 patients completed the study after 3 weeks.
1b-v) CONCLUSIONS/DISCUSSION in abstract for negative trials
Our trial was concluded as positive results

INTRODUCTION

2a-i) Problem and the type of system/solution
These side effects may cause poor drug compliance, prohibiting successful anticancer treatment. Poor education is one of the main determinants of poor adherence to chemotherapy. Therefore, proper education contribute to improved clinical outcomes
2a-ii) Scientific background, rationale: What is known about the (type of) system
Recent studies with health-related internet games have shown positive effects, such as improving coping strategies for health problems
Does your paper address CONSORT subitem 2b?
We hypothesized that mobile gaming would lead to increased drug compliance, decreased physical side effects of chemotherapy, and improved psychological status among patients.

METHODS

3a) CONSORT: Description of trial design (such as parallel, factorial) including allocation ratio
A flow diagram of the study is shown in Figure 2.
3b) CONSORT: Important changes to methods after trial commencement (such as eligibility criteria), with reasons
We inserted sentences
3b-i) Bug fixes, Downtimes, Content Changes
Our study was very short term study and this address was not in the ms.
4a) CONSORT: Eligibility criteria for participants
We address the CONSORT
4a-i) Computer / Internet literacy
The exclusion criteria were as follows
4a-ii) Open vs. closed, web-based vs. face-to-face assessments:
We The patients were unblinded
4a-iii) Information giving during recruitment
Informed consent was obtained from all patients during hospitalization for chemotherapy after explaining the design, protocol
4b) CONSORT: Settings and locations where the data were collected
We explained description as follow up of cell-phone
4b-i) Report if outcomes were (self-)assessed through online questionnaires
The questions were assessed using a self-reported scale of 10
levels with 10 indicating
4b-ii) Report how institutional affiliations are displayed

5) CONSORT: Describe the interventions for each group with sufficient details to allow replication, including how and when they were actually administered
5-i) Mention names, credential, affiliations of the developers, sponsors, and owners
A mobile game, ILOVEBREAST (CLGAMES, Seoul, Korea) was developed
5-ii) Describe the history/development process

5-iii) Revisions and updating

5-iv) Quality assurance methods

5-v) Ensure replicability by publishing the source code, and/or providing screenshots/screen-capture video, and/or providing flowcharts of the algorithms used
Representative screenshots of the ILOVEBREAST game.
5-vi) Digital preservation

5-vii) Access
For patients in the game group, the study mobile game (ILOVEBREAST) was installed on the participants' smartphones
5-viii) Mode of delivery, features/functionalities/components of the intervention and comparator, and the theoretical framework
They were interviewed every week via cell phone until the end of the study
5-ix) Describe use parameters
They were recommended to play the game for >30 minutes a day, 3 times per week
5-x) Clarify the level of human involvement

5-xi) Report any prompts/reminders used
They were interviewed every week via cell phone until the end of the study
5-xii) Describe any co-interventions (incl. training/support)
They were interviewed every week via cell phone until the end of the study
6a) CONSORT: Completely defined pre-specified primary and secondary outcome measures, including how and when they were assessed
The game group also showed improved compliance to medications compared with the control group (K-MARS score)
6a-i) Online questionnaires: describe if they were validated for online use and apply CHERRIES items to describe how the questionnaires were designed/deployed

6a-ii) Describe whether and how "use" (including intensity of use/dosage) was defined/measured/monitored
The time spent on game playing in the mobile game group was higher than that spent for self-education in the control group
6a-iii) Describe whether, how, and when qualitative feedback from participants was obtained
The patients in the game group were requested to assess their level of satisfaction with ILOVEBREAST

**6b) CONSORT: Any changes to trial outcomes after the trial commenced, with reasons**		
We explained description as follow up of cell-phone		
**7a) CONSORT: How sample size was determined**		
**7a-i) Describe whether and how expected attrition was taken into account when calculating the sample size**		
Because this study was a proof-of-concept trial, the sample size calculation was done on a practical basis		
**7b) CONSORT: When applicable, explanation of any interim analyses and stopping guidelines**		
The game group also showed improved compliance to medications compared with the control group (K-MARS score)		
**8a) CONSORT: Method used to generate the random allocation sequence**		
Our participants were allocated with random number generation		
**8b) CONSORT: Type of randomisation; details of any restriction (such as blocking and block size)**		
We use simple randomization		
**9) CONSORT: Mechanism used to implement the random allocation sequence (such as sequentially numbered containers), describing any steps taken to conceal the sequence until interventions were assigned**		
utilizing an interactive Web randomization system.		
**10) CONSORT: Who generated the random allocation sequence, who enrolled participants, and who assigned participants to interventions**		
The statistician who was independent department performed randomization		
**11a) CONSORT: Blinding - If done, who was blinded after assignment to interventions (for example, participants, care providers, those assessing outcomes) and how**		
**11a-i) Specify who was blinded, and who wasn't**		
Our study was devided into two groups, not blinded		
**11a-ii) Discuss e.g., whether participants knew which intervention was the "intervention of interest" and which one was the "comparator"**		
Patients were randomly assigned to education using mobile game play (game group) or conventional education (control group)		
**11b) CONSORT: If relevant, description of the similarity of interventions**		
These are also education about toxicities of chemotherapy		
**12a) CONSORT: Statistical methods used to compare groups for primary and secondary outcomes**		
Medication Adherence Rating Scale (K-MARS), which has a Cronbach reliability alpha of .71		
**12a-i) Imputation techniques to deal with attrition / missing values**		
Among them, 2 were excluded because of severe depressive and anxiety symptoms, 4 for having difficulties in using the mobile game, and 1 for withdrawal of consent for an unspecified reason.ntrol group)		
**12b) CONSORT: Methods for additional analyses, such as subgroup analyses and adjusted analyses**		
Continuous variables were compared with independent t tests<br>or Mann-Whitney U-tests as appropriate. The Chi-square test<br>or Fisher's exact test was used for dichotomous variables		
RESULTS		
**13a) CONSORT: For each group, the numbers of participants who were randomly assigned, received intended treatment, and were analysed for the primary outcome**		
Among the 76 female patients who were finally enrolled, 36 and 40 were randomly assigned to the mobile game and control groups, respectively		
**13b) CONSORT: For each group, losses and exclusions after randomisation, together with reasons**		
Among them, 2 were excluded because of severe depressive and anxiety symptoms, 4 for having difficulties in using the mobile game, and 1 for withdrawal of consent		
**13b-i) Attrition diagram**		
**14a) CONSORT: Dates defining the periods of recruitment and follow-up**		
Patients with pathologically proven, clinical stage IV breast cancer were enrolled in this study at Chung-Ang University Hospital, Korea, from September 2013 to September 2014		
**14a-i) Indicate if critical "secular events" fell into the study period**		
**14b) CONSORT: Why the trial ended or was stopped (early)**		
Our study was finished after the 3-weeks follow-up of 76 female patients.		
**15) CONSORT: A table showing baseline demographic and clinical characteristics for each group**		
The baseline characteristics of the study subjects are summarized in Table 1.		
**15-i) Report demographics associated with digital divide issues**		
Because of easy-to-use Web-based game, beginners were not different from experienced participants		
**16a) CONSORT: For each group, number of participants (denominator) included in each analysis and whether the analysis was by original assigned groups**		
**16-i) Report multiple "denominators" and provide definitions**		
Our analysis was performed with number of participants (denominator) included in each analysis		
**16-ii) Primary analysis should be intent-to-treat**		
**17a) CONSORT: For each primary and secondary outcome, results for each group, and the estimated effect size and its precision (such as 95% confidence interval)**		
the significance level alpha was set at.05,		
**17a-i) Presentation of process outcomes such as metrics of use and intensity of use**		
**17b) CONSORT: For binary outcomes, presentation of both absolute and relative effect sizes is recommended**		
We didn't use the binary outcomes		
**18) CONSORT: Results of any other analyses performed, including subgroup analyses and adjusted analyses, distinguishing pre-specified from exploratory**		
We didn't use subgroup analyses and adjusted analyses		
**18-i) Subgroup analysis of comparing only users**		
**19) CONSORT: All important harms or unintended effects in each group**		
Our study didn't occurred SAE. Also, unintended effects were not observed		
**19-i) Include privacy breaches, technical problems**		
**19-ii) Include qualitative feedback from participants or observations from staff/researchers**		
DISCUSSION		
**20) CONSORT: Trial limitations, addressing sources of potential bias, imprecision, multiplicity of analyses**		
**20-i) Typical limitations in ehealth trials**		
The major limitation of this study is the small sample size and the short study period.		
**21) CONSORT: Generalisability (external validity, applicability) of the trial findings**		
**21-i) Generalizability to other populations**		
**21-ii) Discuss if there were elements in the RCT that would be different in a routine application setting**		
**22) CONSORT: Interpretation consistent with results, balancing benefits and harms, and considering other relevant evidence**		
**22-i) Restate study questions and summarize the answers suggested by the data, starting with primary outcomes and process outcomes (use)**		
The mobile game ILOVEBREAST was developed to help patients with advanced breast cancer learn more about the disease course, properties of medications, and expected adverse drug reactions.		
**22-ii) Highlight unanswered new questions, suggest future research**		
We believe this fact provides an important lesson for future developers.		
Other information		
**23) CONSORT: Registration number and name of trial registry**		
NCT03205969		
**24) CONSORT: Where the full trial protocol can be accessed, if available**		
We summarized the trial protocol at "Study Procedure" section		

**25) CONSORT: Sources of funding and other support (such as supply of drugs), role of funders**		
This research study was supported by a grant of Nexon 2014 and a grant from the Korea Creative Content Agency, Ministry of Culture, Sports and Tourism (2013040436).		
**X26-i) Comment on ethics committee approval**		
The Chung-Ang University Hospital Institutional Review Board approved the research protocol for this study (Number C20141447)		
**x26-ii) Outline informed consent procedures**		
Informed consent was obtained from all patients during hospitalization for chemotherapy after explaining the protocol, and consequences of the study.		
**X26-iii) Safety and security procedures**		
**X27-i) State the relation of the study team towards the system being evaluated**		
